# A dominant variant in apoptosis-related gene *XKR8* is relevant to hereditary auditory neuropathy

**DOI:** 10.1186/s12967-023-04139-x

**Published:** 2023-04-26

**Authors:** Kaitian Chen, Changwu Li, Chang Dong, Xiaoqing Cen, Yueying Wang, Yue Liang, Yuanping Zhu, Shubin Fang, Hongyan Jiang

**Affiliations:** 1 Department of Otorhinolaryngology, The First Affiliated Hospital, Sun Yat-sen University, Guangzhou, 510080 Guangdong People’s Republic of China; 2 Institute of Otorhinolaryngology, Sun Yat-sen University, Guangzhou, 510080 Guangdong People’s Republic of China; 3 Department of Otorhinolaryngology, Hainan General Hospital, Haikou, 570311 Hainan China

**Keywords:** Auditory neuropathy, Gene, Hearing loss, Variant, XKR8

## Abstract

**Background:**

Auditory neuropathy is an unusual type of hearing loss. At least 40% of patients with this disease have underlying genetic causes. However, in many hereditary auditory neuropathy cases, etiology remains undetermined.

**Methods:**

We collected data and blood samples from a four-generation Chinese family. After excluding relevant variants in known deafness-related genes, exome sequencing was conducted. Candidate genes were verified by pedigree segregation, transcript/protein expression in the mouse cochlea, and plasmid expression studies in HEK 293T cells. Moreover, a mutant mouse model was generated and underwent hearing evaluations; protein localization in the inner ear was also assessed.

**Results:**

The clinical features of the family were diagnosed as auditory neuropathy. A novel variant c.710G > A (p.W237X) in apoptosis-related gene *XKR8* was identified. Genotyping of 16 family members confirmed the segregation of this variant with the deafness phenotype. Both *XKR8* mRNA and XKR8 protein were expressed in the mouse inner ear, predominantly in regions of spiral ganglion neurons; Moreover, this nonsense variant impaired the surface localization of XKR8 in cells. Transgenic mutant mice exhibited late-onset auditory neuropathy, and their altered XKR8 protein localization in the inner ear confirmed the damaging effects of this variant.

**Conclusions:**

We identified a variant in the *XKR8* gene that is relevant to auditory neuropathy. The essential role of *XKR8* in inner ear development and neural homeostasis should be explored.

**Supplementary Information:**

The online version contains supplementary material available at 10.1186/s12967-023-04139-x.

## Introduction

Hearing loss is usually associated with a loss of hair cell function, which can be fully restored by cochlear implantation. In contrast, auditory neuropathy (AN), also referred to as auditory neuropathy spectrum disorder, results from synaptic disorders in afferent nerves with intact outer hair cell function [1]. In AN, pathological changes in any part of the auditory nerve can disturb the synchronism of binaural processing, which greatly affects quality of life in patients.

It is estimated that about 40% of AN has a genetic origin [2]. The otoferlin (*OTOF*) gene is the most well-studied AN-related gene [3]. Variants in this gene have been found in patients from different countries and populations. To date, several other genes related to AN have been identified, such as *DIAPH3* [4] and *PJVK* [5]. However, the genetic etiology in many patients with “hereditary” AN is yet to be revealed.

The *XKR8* (NM_018053) gene is located at 1p35.3, and has a total length of 2710 bp and a coding region of 1188 bp. It encodes XK-related protein 8 (XKR8), which contains 395 amino acids and 10 transmembrane regions and is localized at the cell membrane surface. This protein belongs to the XK-related protein family (XKR1–XKR9) [6]. Suzuki et al. reported that XKR8 can promote phosphatidylserine (PtdSer) to turn outward to the cell membrane and mediate apoptosis [6]. Thus far, studies of *XKR8* have focused on cell apoptosis; there are no reports of a relationship between *XKR8* and the inner ear or deafness.

In the present study, we report the identification of *XKR8* as an AN-related gene in a Chinese family. A combination of clinical data, genetic verification, and functional analysis in mice and cells supports our hypothesis that a dominant variant in *XKR8* is linked to hereditary AN in this family.

## Methods

### Informed consent and ethical considerations

Informed consent was obtained from all patients or their guardians. This study was approved by the institutional review board of the First Affiliated Hospital, Sun Yat-sen University. All animal experiments were performed using protocols approved by the Animal Care and Use Committee of Sun Yat-sen University.

### Patients and data collection

**Fig. 1:**
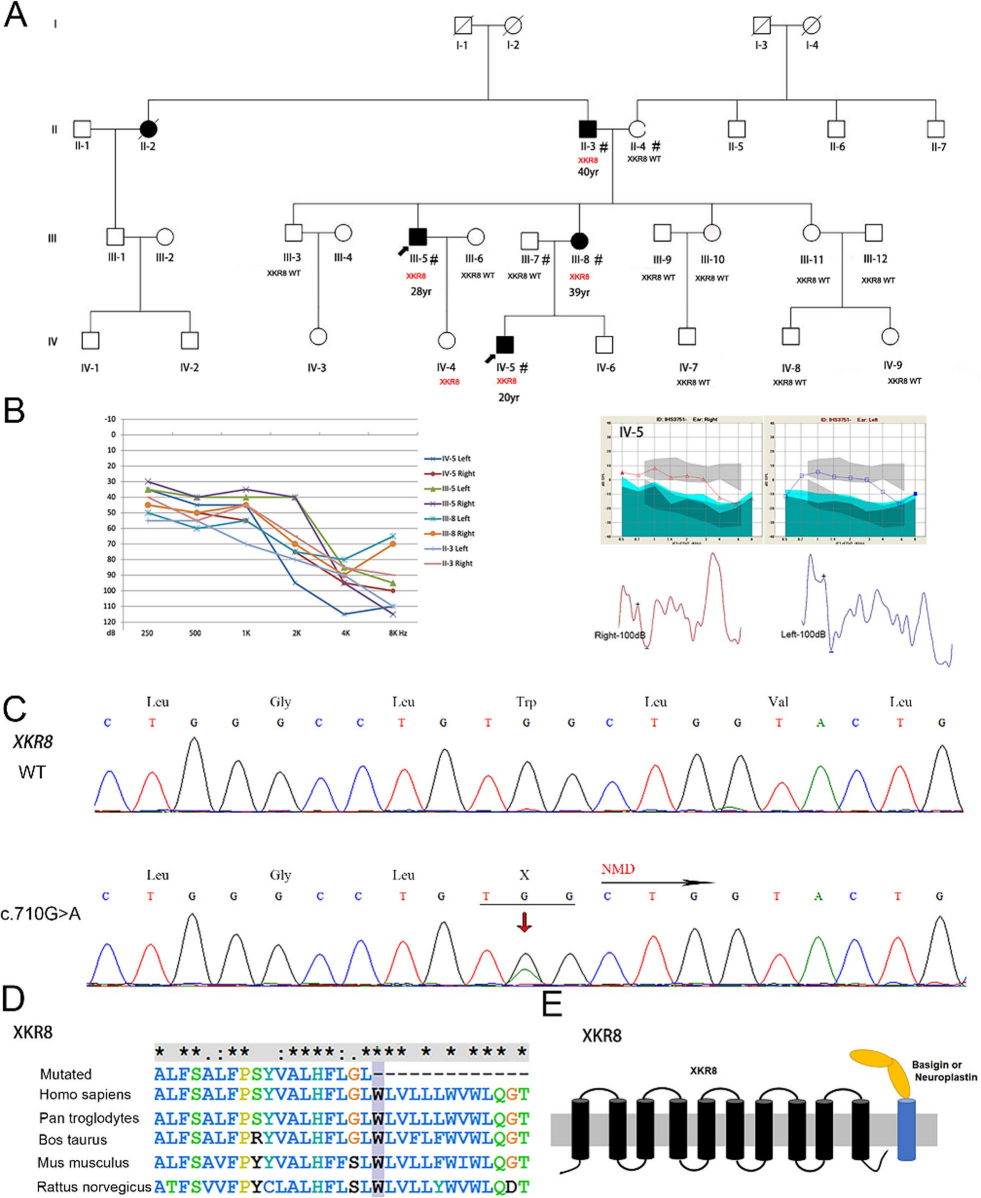
Pedigree and hearing of the family with AN are shown (**A** and **B**). # Indicates members selected for exome sequencing. The onset of deafness is shown for each patient. *XKR8* in red indicates heterozygotes. Note that IV-4 (9 years old) had normal hearing at the time of analysis. Characteristic hearing data (from IV-5) is shown, with impaired speech perception scores (56% and 53% in the left and right ears, respectively) and normal OAE. Exome sequencing identified a novel variant in *XKR8* (**C**), which is highly conserved (**D**).  The protein structure of XKR8 [13] is shown (**E**)

A four-generation family with postlingual hearing loss from Guangxi Province, China, visited our clinic for genetic counseling (Fig. 1). The probands (III-5 and IV-5) underwent a complete medical history and physical examination, which excluded a non-genetic etiology. Age-appropriate audiological examinations were performed, including pure-tone audiometry and/or auditory brainstem response (ABR) and distortion product otoacoustic emissions (DPOAE) evaluations. Speech perception scores were also evaluated. Temporal computed tomography examinations were performed to rule out the possibility of inner ear malformations. In addition, syndromic symptoms other than hearing loss in the other two patients (II-3 and III-8) were eliminated using medical records.

### Molecular screening of known variants

Blood samples were collected from 16 members of this family (Fig. 1), and DNA isolation was performed using QuickGene 610 L (KURABO, Osaka, Japan) and the matching DNA blood kit (DB-L; KURABO). Targeted sequences of common genes (e.g., *GJB2*, *SLC26A4*, and mitochondrial genes) were first sequenced as previously described [7]. In addition, a customized panel (xGen; Integrated DNA Technologies, Coralville, IA, USA) comprising 127 known deafness genes [8] was used in proband IV-5 because of the negative results of common deafness genes.

### Exome sequencing and segregation analysis

Exome sequencing was performed in four affected (II-3, III-5, III-8, and IV-5) and two unaffected (II-4 and III-7) family members (marked with # in Fig. 1A). Briefly, DNA was amplified by ligation-mediated polymerase chain reaction (PCR) before being purified and hybridized to the SureSelect Biotinylated RNA Library (Agilent, Santa Clara, CA, USA) for enrichment. Captured ligation-mediated PCR products were analyzed using the Agilent 2100 Bioanalyzer (Agilent). Each captured library was then loaded onto the HiSeq 2000 platform (Illumina, San Diego, USA). Raw image files were processed using Illumina base-calling Software 1.7 for base-calling with default parameters, and the sequences of each individual were generated as 90-/100-bp pair-end reads. Burrows-Wheeler Aligner was used to perform raw data alignment. Single nucleotide polymorphisms were identified using SOAPsnp, SAMtools, or GATK. AnnoDB software was used in-house to annotate the likely variant results.

Candidate pathogenic variants were classified as nonsense, missense, splice-site, or indel variants. Variant pathogenicity was assessed by in silico analysis [8]. All sequences were aligned and compared with published sequences from the National Center for Biotechnology Information database. All variants were categorized according to the American College of Medical Genetics and Genomics/Association for Molecular Pathology guidelines [9, 10].

After standard data filtering, the target sequences in candidate genes were subjected to Sanger sequencing in 16 family members. Amino acid conservation alignment across different *XKR8* gene families (*Bos taurus*: XP_002685733.1; *Mus musculus*: NP_958756.1; *Rattus norvegicus*: NP_001012099.1; *Homo sapiens*: NP_060523.2; *Pan troglodytes*: NP_001028209.1) was completed using ClustalX2 software. The transcript levels of candidate genes in adult mouse cochlea were evaluated by reverse transcription PCR.

### Mouse sample collection and cell culture

C57BL mice were used in all experiments. Transgenic mice carrying heterozygous variant c.710G > A (referred as *XKR8*^+/−^) were generated from the Jikai Laboratory (Shanghai, China). Genotypic verification of the transgenic mice was performed according to recommendations from the Jikai Laboratory. For mRNA detection, samples of cochlea were dissolved in TRIzol. The rest of each cochlea was immediately fixed with 4% paraformaldehyde for at least 1 h at room temperature. Frozen cochlear sections were used for immunofluorescence staining.

HKE 239T cells were cultured with 10% fetal bovine serum/Dulbecco’s Modified Eagle’s Medium at 37 °C in a CO_2_ incubator for transfection. One day before transfection, 0.5–2 × 10^5^ cells were plated in 500 µL of growth medium without antibiotics to ensure 90–95% confluence at the time of transfection.

### RNA isolation and reverse transcription PCR of the mouse cochlea

A routine TRIzol method was used for the isolation of postnatal day 30 mouse cochlear RNA; the product was converted to cDNA using the All-in-One First-Strand cDNA Synthesis Kit (GeneCopoeia, Rockville, MD, USA). Forward and reverse primer pairs for mouse *XKR8* were designed (F-TCTTCCCTCCAAGCCACG; R-ACACGCAAAGCCAGTCCC), resulting in a 444-bp PCR product. The *GAPDH* gene was also amplified (F- AACGGGAAGCCCATCACC; R-CAGCCTTGGCAGCACCAG). The resulting products were detected by 2% agarose gel electrophoresis. Data were analyzed using ImageJ software (National Institutes of Health, Bethesda, MD, USA). Each experiment was replicated at least three times.

### Immunofluorescence of XKR8 protein

Staining of XKR8 was performed in cross sections of mouse cochlea. Sections were blocked with 1% bovine serum albumin (BSA) and 1% Triton X-100 in 0.1 M phosphate buffer (pH 7.2) for 1 h at room temperature. Next, the sections were incubated overnight at 4 °C with primary antibodies against XKR8 (Santa Cruz Biotechnology, Santa Cruz, CA, USA), myosin VIIA (MYO7A; Abcam, Cambridge, UK), and class III beta-tubulin (TUJ1; Abcam) diluted in 1% BSA and 10% Triton X-100. Sections were then incubated for 1 h at room temperature with secondary antibodies diluted in 0.1% BSA and 0.1% Triton X-100. An LSM 700 confocal microscope (Ziess, Oberkochen, Germany) or Leica microscope (Wetzlar, Germany) was used to capture images.

### Plasmid construction and transfection into human embryonic kidney (HEK) 293T cells

The fluorescent plasmids pEGFP-C2 and pmCherry-C1 were obtained from our laboratory. The entire coding sequences of human wild-type (WT) *XKR8* and the *XKR8* c.710G > A mutant were inserted into the plasmids. Four types of recombined plasmids were constructed and confirmed by Sanger sequencing: pEGFP-C2-*XKR8*-c.710G-A, pEGFP-C2-*XKR8*-WT, pmCherry-C1-*XKR8*-c.710G-A, and pmCherry-C1-*XKR8*-WT.

Plasmids were then transfected into HEK 293T cells using Lipofectamine 2000 Transfection Reagent (Invitrogen, Waltham, MA, USA). For the heterozygous model, WT and mutant plasmids were mixed at a 1:1 ratio and added to the cell plate. Cells were maintained at 37 °C in a CO_2_ incubator for 48–72 h prior to immunofluorescence. Each experiment was replicated at least three times.

### Mouse ABR and DPOAE

Mouse ABR was conducted using a High Frequency Transducer (Intelligent Hearing Systems, Miami, FL, USA). The computer generated 4, 8, 16, and 32 kHz click and tone burst sounds as the stimuli, with the following settings: duration of acoustic stimulation, 5 ms; rising and falling time, 0.5 ms; scanning time, 10 ms; stimulation sound repetition rate, 11 times/s, band-pass filtering frequency, 300–3000 Hz; acquisition amplification gain, 100,000 times; and average stacking, 1024 times. Data were analyzed using SmartEP 5.40 software. In mice, DPOAE evaluations were performed using DP-Gram on a Titan system (Interacoustics, Middelfart, Denmark) in our lab according to the manufacturer’s instructions.

### Statistical analysis

IBM SPSS Statistics for Windows, version 23.0 (IBM Corp., Armonk, NY, USA) was used for statistical analyses. All data are presented as the mean ± standard deviation. Two-tailed, independent Student’s t-tests were performed. P-values < 0.05 were considered significant.

## Results

### Clinical data of the family with AN

This family contained four patients living with hearing loss (Fig. 1A). The affected members (III-3, III-5, III-8, and IV-5) developed hearing loss between the second and fourth decades of life. In all patients, outer hair cell function as measured by OAE was preserved but speech perception scores were decreased (by 20–60%). The hearing levels of the patients are summarized in Fig. 1B. The presence of normal OAE and abnormal ABR results suggested the possibility of auditory nerve/synapse lesions, which is consistent with dominant AN.

### Exome sequencing identifies a heterozygous variant in *XKR8*

Genetic tests for common deafness genes (e.g., *GJB2* or *SLC26A4*) and the customized gene panel failed to identify any causal variant in the family. We therefore performed exome sequencing, in which 46 variants in 45 genes were filtered (Additional file 1: Table S1). The transcript levels of these 45 genes in adult mice cochlea were analyzed (data not shown); 21 and 6 genes were strongly and weakly expressed in the cochlea, respectively. Sanger sequencing of these variants was then performed in peripheral blood samples from the family. A heterozygous nonsense mutation, c.710G > A (p. W237X, exon 3) in *XKR8*, was found to exclusively co-segregate with the AN phenotype in this family (Fig. 1C).

The novel mutation c.710G > A was predicted to be deleterious by computational tools. It introduces a premature stop codon at Trp237, leading to nonsense-mediated mRNA decay (NMD) or the production of a truncated XKR8 protein. The amino acids around Trp237 are highly conserved among different species (Fig. 1D). A schematic structure of full length XKR8 is shown in Fig. 1E.

###  *XKR8* is expressed in the mouse inner ear

**Fig. 2:**
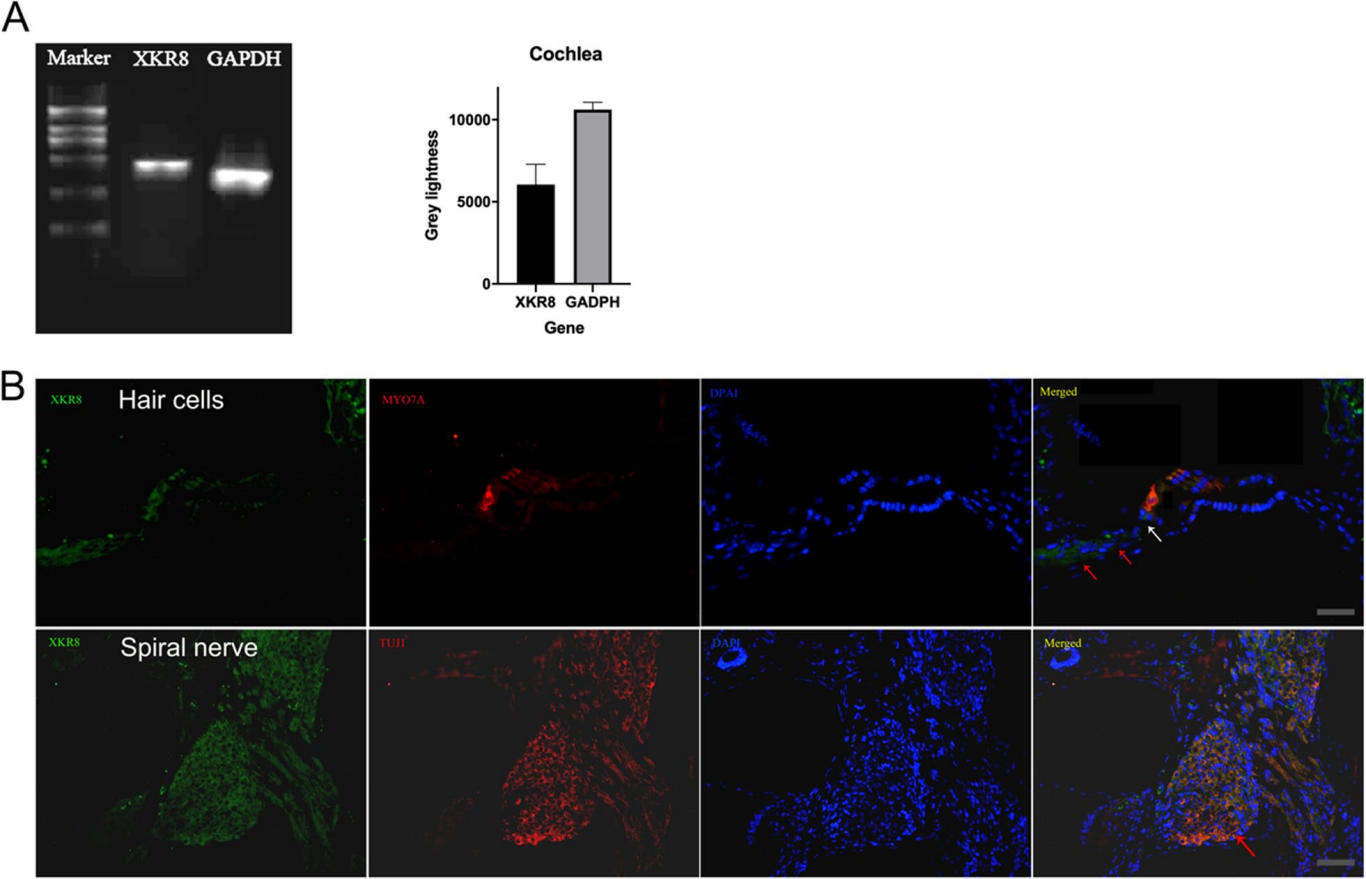
The expression of *XKR8* mRNA in the adult cochlea was confirmed by reverse transcription PCR (**A**). The ratio of *XKR8*/*GAPDH* was 0.572 ± 0.122 (n = 3). Representative results of immunofluorescence staining in the cochlea of adult mice (**B**). XKR8 protein was weakly expressed at the bottom of inner hair cells (white arrow) and strongly expressed in auditory neurons (red arrows). Scar bar = 100 μm

The *XKR8* gene is reportedly widely expressed in many organs, including the liver, brain, and placenta [6]. However, little is known about its expression in the inner ear. Because hearing loss occurred in adulthood in this family, we hypothesized that the XKR8 protein may be necessary for cochlear function in adults. As shown in Fig. 2A, *XKR8* was stably present in the postnatal day 30 cochlea in mice. The average ratio of *XKR8* to *GAPDH* transcript density was 0.572 ± 0.122 (n = 3).

Immunofluorescence suggested that XKR8 was weakly expressed at the bottom of inner hair cells but strongly expressed in auditory neurons along the cochlear modiolus. XKR8 was co-localized with TUJ1 and projected from ribbon synapses along the spiral limbus, similar to the location of type I afferent nerve fibers. In contrast, XKR8-positive fluorescence was not observed in hair cells or supporting cells (Fig. 2B). This observation is consistent with the pathophysiological features of AN.

### The *XKR8* c.710G > A mutation disrupts the surface localization of XKR8

**Fig. 3:**
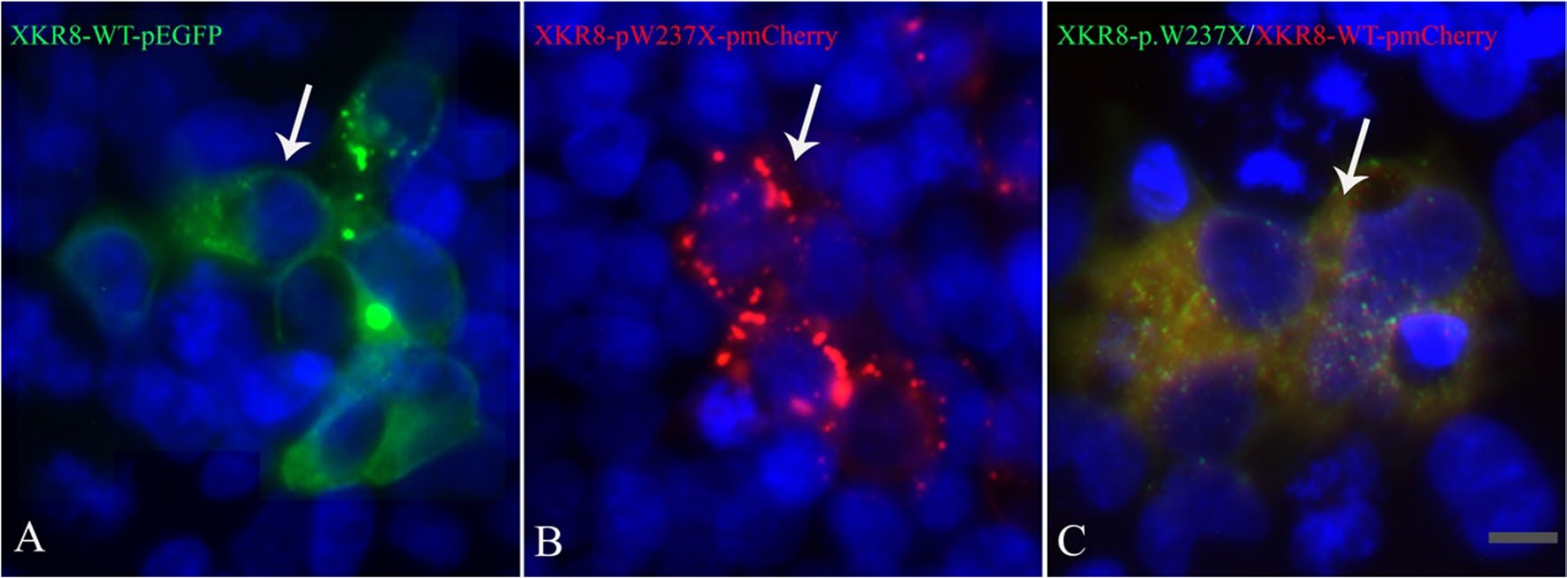
WT XKR8 was expressed at the surface of HEK 293T cell membranes (white arrow; **A**). Homozygous (**B**) and heterozygous (**C**) variants resulted in a lack of morphological XKR8 protein at the cell membrane surface, and large plaques were observed around nuclei (white arrows; B and C). Representative results are shown. Nuclei were stained with 4′,6-diamidino-2-phenylindole (DAPI; blue). Scar bar = 10 μm

In WT mice, XKR8 was localized at the surface of cell membranes. By contrast, in mice with homozygous or heterozygous variants, the normal protein structure did not form at the cell membrane surface (Fig. 3). Instead, large plaques were observed in cell cytoplasm, suggesting that this variant may exert a dominant-negative effect.

### *XKR8*^+/−^ mice display delayed onset of hearing loss that resembles AN

**Fig. 4:**
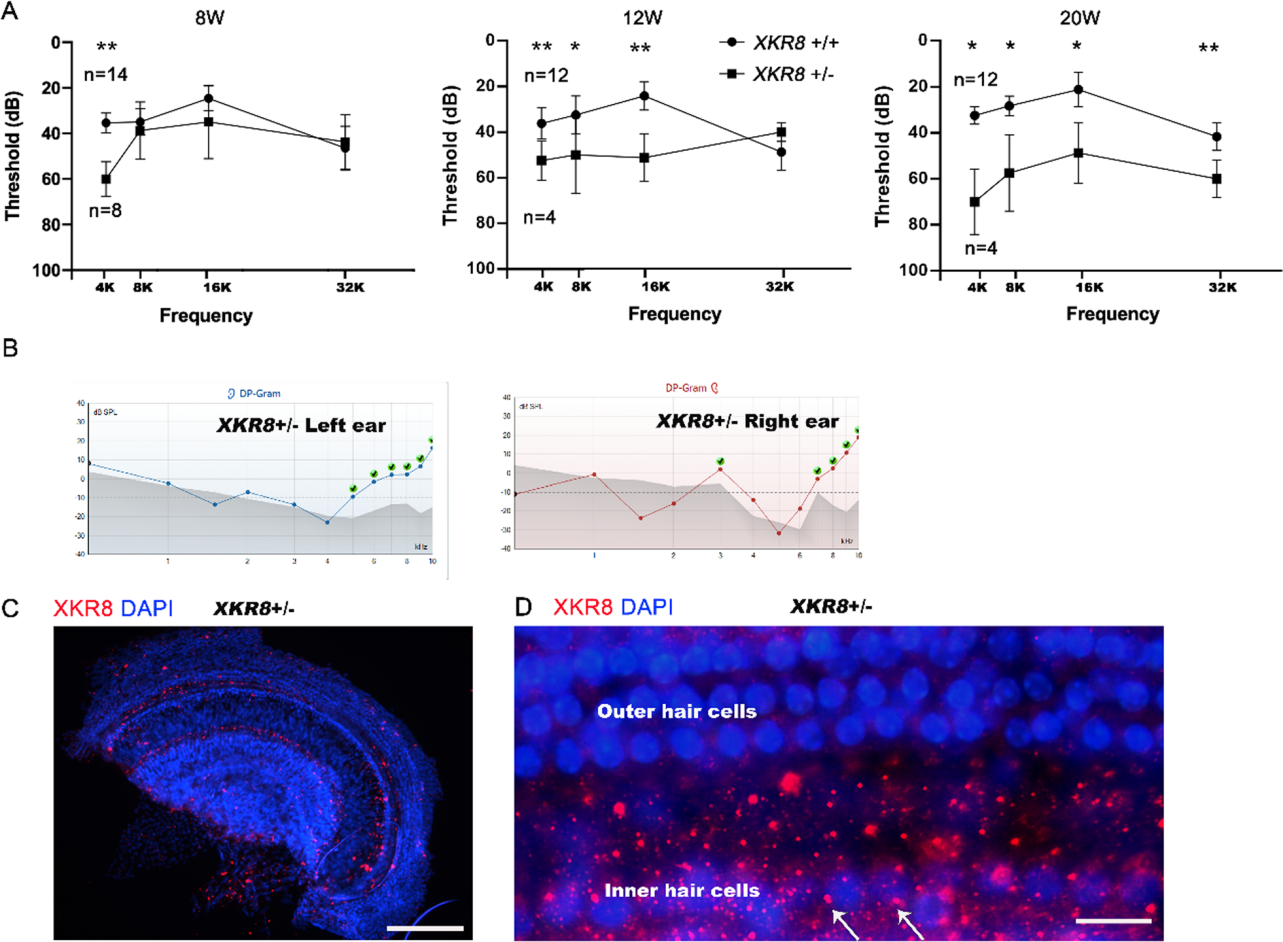
Hearing loss in the *XKR8*^+/−^ mouse model. Compared with WT mice, *XKR8*^+/−^ mice showed hearing loss from the age of 12 weeks (**A**) but had intact hair cell function at 20 weeks of age (**B**). Representative results showed scattered XKR8-positive plaques in the mouse cochlea at 20 weeks of age (**C** and **D**). Truncated XKR8 was localized in regions beneath inner hair cells (white arrows in **D**). *p < 0.05, **p < 0.01 (indicating different hearing results between the two groups). Scale bars: **C**, 500 μm; **D**, 25 μm

In 8-week-old *XKR8*^+/−^ mice, hearing was within the normal range (Fig. 4); however, hearing loss developed and worsened as the mice aged. Compared with the WT group, we observed various degrees of hearing loss in *XKR8*^+/−^ mice at 12 weeks of age. Furthermore, elevated hearing thresholds were more apparent in *XKR8*^+/−^ mice at 20 weeks of age (Fig. 1D). However, OAE were still able to be evoked in 20-week-old mice, suggesting that any impairments were located behind the outer hair cells (Fig. 4B).

Similarly, cochlear basilar membrane staining revealed that XKR8-positive plaques were scattered around the spiral ganglion nerve (Fig. 4C) in 20-week-old *XKR8*^+/−^ mice. In cross sections of the cochlea, truncated XKR8 protein was frequently located in regions beneath inner hair cells and next to spiral ganglion neurons (Fig. 4D).

Collectively, these findings suggest that this *XKR8* variant may be a genetic basis of dominant AN.

## Discussion

In the present study, we identified a novel AN-related gene, *XKR8*, which is probably involved in inner ear development and neural homeostasis. The *XKR8* gene encodes the scramblase protein XKR8, which promotes PtdSer exposure on the cell surface during apoptosis [6, 11]. Additional physiological functions of XKR8 include involvement in the engulfment of apoptotic cells and the regulation of myoblast differentiation [12, 13]. Apoptotic stimuli do not induce PtdSer exposure in *XKR8*-null germ cells, which leads to infertility in male mice [14]. Furthermore, impaired XKR8-mediated PtdSer exposure in apoptotic lymphocytes and aged neutrophils activates the immune system, causing lupus-like autoimmune disease in mice [15]. Although *XKR8* has been extensively studied in many organs and cells, its role in hearing has not previously been reported.

A sensitivity or resistance to mRNA degradation by NMD depends on the exon position of a premature stop codon [16]. The c.710G > A mutation encodes a premature termination at Trp237 that was frequently subject to degradation by activating the NMD surveillance pathway; it is likely to be pathogenic according to variant pathogenicity guidelines [9, 10]. Any remaining truncated protein that escapes NMD may impair the normal function of XKR8 protein through a dominant effect, as observed in our cell transfection tests.

The location of XKR8 in spiral ganglion cells and the bottom of the inner ear led us to speculate that *XKR8* has an essential role in the cochlea. XKR8 can form a molecular complex with basigin (BSG) or neuroplastin (NPTN), which are type I membrane proteins from the immunoglobin superfamily [17]. Intracellular XKR8 fails to expose cellular PtdSer after apoptotic stimuli in the absence of BSG and NPTN, suggesting that the scrambling activity of XKR8 may require the chaperone molecules BSG/NPTN at the plasma membrane [17]. Furthermore, mice deficient in neuroplastin 65 (NP65), a neuronal and synapse-enriched glycoprotein, present early-onset hearing loss with impaired synaptogenesis in inner hair cells. NP65 is exclusively localized in the presynaptic region of inner hair cells [18]; this partly overlaps with the XKR8 expression observed in our study. BSG has also been detected in the mouse cochlea [19]. Although the exact pathomechanism of *XKR8* in AN is yet to be clarified, a molecular correlation among XKR8, NP65, and BSG strongly implicates an essential role of *XKR8* in maintaining synaptic homeostasis and neural function in the cochlea. Moreover, dysregulated *XKR8* expression may impair BSG or NPTN function in synaptogenesis or the spiral ganglion.

To confirm our results from the family with AN, we generated *XKR8*^+/−^ mutant mice. Hearing in these mice remained stable after the onset of hearing, and our primary animal hearing data were consistent with clinical observations from the four patients in this family. An elevated hearing threshold may be associated with a partial loss of function in XKR8 protein, as observed in the 20-week-old mutant mice. Disrupted synaptogenesis and neural function may in part, or in combination with apoptotic dysfunction in the inner ear, contribute to the AN phenotype observed in both the patients and the mouse model. We are currently investigating the detailed pathomechanism of this *XKR8* variant in transgenic mouse.

Our study has some limitations. The physiological function of *XKR8* in the inner ear requires further studies. Additionally, the pathomechanism of the identified *XKR8* mutation remains unclear. Finally, the prevalence of the *XKR8* variant in AN and nonsyndromic hearing loss also needs to be investigated.

## Conclusions

We provided evidence that a variant in the *XKR8* gene is responsible for autosomal dominant AN in this family. The essential role of *XKR8* in inner ear development and neural homeostasis should be further investigated.

## Supplementary Information

**Additional file 1.** Table S1.

## Data Availability

The data that support the findings of this study are available from the corresponding author upon reasonable request.
